# Identification of patients at high risk of negative psychological consequences associated with BRCA1 mutation

**DOI:** 10.1186/1897-4287-10-S1-A14

**Published:** 2012-01-12

**Authors:** S Ertmański, K Metcalfe, J Trempała, M Głowacka, J Lubiński, S Narod, J Gronwald

**Affiliations:** 1 International Hereditary Cancer Center, Pomeranian Medical University, Szczecin, Poland; 2 Lawrence S. Bloomberg Faculty of Nursing, University of Toronto, Toronto, Canada; 3 Department of Psychology of Human Development, Kazimierz Wielki University in Bydgoszcz, Bydgoszcz, Poland; 4 Department of Health Sciences, Poznań University of Medical Sciences, Poznań, Poland; 5 Women’s College Research Institute, University of Toronto, Toronto, Canada

## 

Genetic studies of hereditary predisposition to cancer are becoming more accessible and acceptable form of cancer prevention. However, it is suggested that there is a potential impact of diagnosed high risk of developing cancer on the psychological functioning of patients. Research on this subject has been carried out in other countries, while in Poland similar studies using psychological tests have not been conducted so far. The objectives of the research were:

1. psychological assessment of patients before undergoing of genetic test diagnosing high predisposition to breast cancer or ovarian cancer (BRCA1 test),

2. assessment of psychological reactions of patients after receiving of the positive BRCA1 test result,

3. evaluation of the usefulness of selected psychological tests for identification of patients likely to respond by excessive psychological distress to the positive BRCA1 test result.

It was found that the average level of trait anxiety among BRCA1 carriers before receiving the genetic test result was 5.6, and the level of basic hope - 6.0 and these values are within the population standards for these tests. The average level of state anxiety was only slightly above normal - 6.7. There were no differences between female patients suffering from cancer and healthy ones. The average level of state anxiety measured one month after receiving the genetic test result increases slightly and after a year - drops slightly below the state before the genetic test - both in groups of people suffering from cancer, and healthy ones (although not statistically significant). The negative psychological reaction to an abnormal genetic test result measured by the IES test (post-test1 after one month) is significantly higher in patients with diagnosed cancer compared to healthy subjects (p=0.0008). The high level of state anxiety (standard ten: 9 and 10) measured prior to obtaining genetic test result was found in 18% of BRCA1 mutation carriers. Statistically significant correlation has been registered between the level of state anxiety and trait anxiety measured prior to obtaining genetic test result and the level of state anxiety measured after one month (p=0.00001) and after one year (p=0.00001) and the level of negative psychological reaction (p=0.0001) measured by the IES test one month after obtaining a genetic test result. Statistically significant negative relationship has been found between the level of basic hope measured before obtaining a genetic test result and the level of state anxiety (p=0.01) and a negative psychological reaction IES (p=0.003) measured one month after obtaining a genetic test result. Such a relationship has not been demonstrated with the level of state anxiety measured after a year. One month after receiving of a genetic test result, 96% of BRCA1 mutation carriers (135/141) recommended genetic testing to other women in a similar situation. By one year posttest, practically all patients recommended genetic testing.

## Conclusions

1. BRCA1 mutation carriers do not exhibit increased constitutional predisposition to excessive negative psychological reactions.

2. There are over ten percent of BRCA1 mutation carriers characterized by a particularly high level of anxiety both before and after undergoing a genetic test that can obtain special benefit from specialist psychological care.

3. The mere fact of genetic testing and obtaining positive genetic BRCA1 test result informing about the predisposition to breast and ovarian cancer does not affect the suffering of long-term anxiety and other negative psychological reactions.

4. Patients affected by cancer have a higher level of negative psychological reaction (IES test after one month) than healthy patients.

5. A result of the measurement of state anxiety by STAI X1 test before undergoing BRCA1 test is a good predictor of psychological reactions observed among BRCA1 mutation carriers after obtaining genetic test result.

6. A result of the measurement of trait anxiety by STAI X2 test before undergoing BRCA1 test is a good predictor of psychological reactions observed among BRCA1 mutation carriers after obtaining genetic test result.

7. A result of the measurement of basic hope by BHI-12 test before undergoing BRCA1 test is a moderately good predictor of psychological reactions observed in the short term (one month) after diagnosing a positive genetic test result, but does not fulfill this role in the long term (over one year).

8. BRCA1 mutation carriers, both in short and long term, recommend to undergo genetic testing to women in similar situations.

